# Adult Versus Pluripotent Stem Cell-Derived Mesenchymal Stem Cells: The Need for More Precise Nomenclature

**DOI:** 10.1007/s40778-016-0060-6

**Published:** 2016-07-28

**Authors:** Michael D. West, Igor Nasonkin, David Larocca, Karen B. Chapman, Francois Binette, Hal Sternberg

**Affiliations:** 1 BioTime, Inc., 1010 Atlantic Ave., Alameda, CA 94501 USA; 2 ReCyte Therapeutics, 1010 Atlantic Ave, Alameda, CA 94501 USA; 3 OncoCyte Corporation, 1010 Atlantic Ave, Alameda, CA 94501 USA; 4 OrthoCyte Corporation, 1010 Atlantic Ave, Alameda, CA 94501 USA

**Keywords:** Embryonic stem cells, Mesenchymal stem cells, Clonal embryonic progenitor cells, Neural crest, Cartilage, Bone

## Abstract

The complexity of human pluripotent stem cell (hPSC) fate represents both opportunity and challenge. In theory, all somatic cell types can be differentiated from hPSCs, opening the door to many opportunities in transplant medicine. However, such clinical applications require high standards of purity and identity, that challenge many existing protocols. This underscores the need for increasing precision in the description of cell identity during hPSC differentiation. We highlight one salient example, namely, the numerous published reports of hPSC-derived mesenchymal stem cells (MSCs). We suggest that many of these reports likely represent an improper use of certain cluster of differentiation (CD) antigens in defining bone marrow-derived MSCs. Instead, most such hPSC-derived mesenchymal cells are likely a complex mixture of embryonic anlagen, primarily of diverse mesodermal and neural crest origins, making precise identification, reproducible manufacture, and uniform differentiation difficult to achieve. We describe a potential path forward that may provide more precision in nomenclature, and cells with higher purity and identity for potential therapeutic use.

## Introduction

Human pluripotent stem cells (hPSCs), including such cell types as human embryonic stem cell (hESC) lines from biparental or parthenogenetic blastocysts or comparable cells produced by nuclear transfer or induced pluripotent stem cell (iPSC) technology, are being widely utilized for basic research and a small number of human therapeutic trials. In addition to pluripotency, hPSCs have the unique property of unlimited proliferation through the abundant expression of the catalytic component of telomerase that generally maintains them with long and stable telomere length [1]. Since telomere length predicts the replicative capacity of cultured somatic cells [2], hPSCs are seen as a renewable source of diverse human somatic cell types with long proliferative lifespan potential.

In contrast to hPSCs, adult stem cells, such as hematopoietic stem cells (HSCs), neuronal stem cells (NSCs), and mesenchymal stem cells (MSCs), generally possess limited differentiation potential (multipotency) and a finite replicative capacity. This limited scale-up potential commonly leads to the need for continual sourcing of tissue to derive new product lots. In the case of MSCs, there is also a loss of differentiation potential after 5–12 passages in vitro [3, 4]. These challenges have, therefore, led to a search for means of manufacturing the counterparts of these adult stem cell types from hPSCs [5].

### Identity of Adult-Derived MSCs

Bone marrow is the site of definitive hematopoiesis in the adult human. Within the bone marrow are cell subpopulations including blood cell progenitors as well as stromal cells [6, 7]. The stromal cells, designated “mesenchymal stem cells” by Arnold Caplan, have been widely studied primarily for their potential utility in the repair of osteochondral tissues [8], though exploratory investigations have also used them for such diverse applications as in the treatment of diabetes [9], heart disease [10], CNS disorders [11], as well as others. Despite numerous attempts to improve terminology and resolve confusion regarding the similarities and differences of putative MSCs from diverse tissue sites, the term continues to be used for any stromal cell isolate that shows evidence of differentiation to osteochondral and adipose cell fates and displays a defined set of cell surface antigens. The original rationale for referring to all such populations as MSCs is that, by definition, stem cells must be able to self-renew and to differentiate. Thus, if stromal cells from a particular tissue can expand in culture (i.e., self-renew) and differentiate down the osteochondral and adipose lineages, then they were referred to as MSCs. This terminology, however, may prevent recognition of important differences in such cells when derived from diverse tissue types such as adipose, umbilical cord, and bone marrow.

To illumine the rationale behind the use of commonly used antigens in identifying MSCs, it is helpful to recall some of the history of early hematology. Since the bone marrow is the site of definitive hematopoiesis in the adult, early efforts to characterize the progenitors of diverse blood cell lineages such as lymphoid (B and T cells), myeloid (monocytes and granulocytes), and erythroid cells (such as red cell progenitors) utilized cell cloning through colony-forming unit (CFU) assays performed in tissues or in methylcellulose similar to those previously used to isolate and characterize bacterial strains on agar. These blood colony-forming unit assays together with other assays led to the identification of cluster of differentiation (CD) antigens that allowed the lineages to be precisely identified. One such useful antigen was CD45 (lymphocyte common antigen) that was observed to be common to all leucocytes [12]. In contrast to blood cell progenitors, the stromal cells that propagated in cultures from the bone marrow were designated colony-forming unit-fibroblasts (CFU-F). Since CD antigens were an active area of research at the time, CD antigen profiles were sought to distinguish the CFU-F from the blood cell types. CFU-F cells were observed to be CD45- [13] and express antigens not normally expressed on blood cell progenitors such as CD29, CD73, CD90, and CD105 [14, 15]. The International Society for Cellular Therapy has suggested that the term “MSC,” or as they suggest, “multipotent mesenchymal stromal cells,” be applied to cells that express the abovementioned pattern of CD antigens, can be cultivated in adherent cultures, and show the potential to differentiate into at least osteochondral and adipocyte lineages [16].

### Identity of hPSC-Derived Mesenchymal Cells

A growing number of reports suggest that MSCs can be robustly generated from hPSCs [17, 18]. The markers used are generally the same as those used to identify adult-derived cells; however, the reported differentiation protocols vary widely [19]. Although these pluripotent stem cell-derived “MSCs” share classically-defined MSC surface markers such as CD29, CD90, CD73, and CD105, and show some similarities in gene expression profiles, they also display clear functional and expression profile differences that distinguish them from adult MSCs [18]. Given that there are potentially thousands of distinct cell types that can be derived from hPSCs, it is reasonable to inquire whether the putative hPSC-derived MSCs actually correspond to adult-derived MSCs or represent a variety of different, as yet unidentified, embryonic progenitor cell types.

We previously performed a colony-forming unit analysis on human embryonic progenitor (hEP) cells derived from hESC in adherent culture to more precisely identify the various types of progenitor cells that constitute hPSC-derived mesenchyme. hEP cells are cultures of hPSCs-derived cells that have lost markers of pluripotency, but have yet to terminally differentiate. We used transcriptomic analysis of global gene expression data to provide an objective measure of the complexity of a hEP cell library of 202 clones. We performed non-negative matrix factorization (NMF) analysis [20] of the data which revealed that there were 140 distinct cell types contained within the library (stability score; *k* = 140). As evidence of the accuracy of this complexity analysis, the NMF clustering did not split biological or technical replicates, and individual clones showed diverse site-specific homeobox gene markers as well as markers of diverse types of mesoderm, such as lateral plate, intermediate, and paraxial mesoderm, as well as diverse types of neural crest cells corresponding to the NMF clustering.

Of these 140 clonal hEP cell lines, seven have been characterized as cells that, like MSCs, can robustly differentiate into osteochondral lineages as indicated by *COL2A1* expression when differentiated in micromass culture in the presence of TGFβ3 [21••]. We compared typical MSC marker gene expression in a panel of randomly selected clonal hEP lines, a group of adult-derived MSC lines, and a panel of numerous diverse adult epithelial, mesenchymal, and blood cell types. Analysis of *CD29*, *CD73*, *CD90*, and *CD105* gene expression showed that these genes were not only expressed by MSCs, but also by most adult-derived cells including such diverse cell types as endothelial cells, urothelial and retinal pigment epithelial cells, Schwann cells, diverse fibroblast types, and smooth muscle cells from multiple tissues. Originally, these markers were chosen to distinguish MSCs from non-adherent hematopoietic cells in the bone marrow and as expected, they were largely absent from a diverse panel of blood cell types. *CD45*, a marker of hematopoietic cells, however, was expressed only in blood cell types and not in the other cell types tested. Similar patterns of CD antigen expression were also observed by flow cytometry. Therefore, it is reasonable to conclude that the expression of the markers *CD29*, *CD73*, *CD90*, and *CD105*, and the absence of *CD45* expression, are effective markers in distinguishing bone marrow stromal cells from blood cells, but are ineffective in distinguishing MSCs from most other somatic cell types.

Our clonal analysis reveals that while expressing commonly-used MSC markers, only a subset of hPSC-derived embryonic mesenchymal cell types are capable of differentiating into osteochondral (*COL2A1* and *IBSP*-expressing) [21••] and adipogenic (*FABP4*-expressing) cells [22•, 23]. It is, therefore, reasonable to conclude that reports that hPSC-derived heterogeneous cultures of mesenchymal cells are “MSCs” is likely an imprecise and unhelpful use of terminology. Instead, they are a complex mixture of cell types, many of which may express commonly-used MSC markers, but others with very distinct cellular identities and/or cell fate. In such heterogeneous cultures, a subset of the cells capable of expressing adipogenic or chondrogenic markers in the presence of BMPs like TGFβ3 could lead to the incorrect conclusion that the entire MSC marker expressing population had similar potential.

**Fig. 1:**
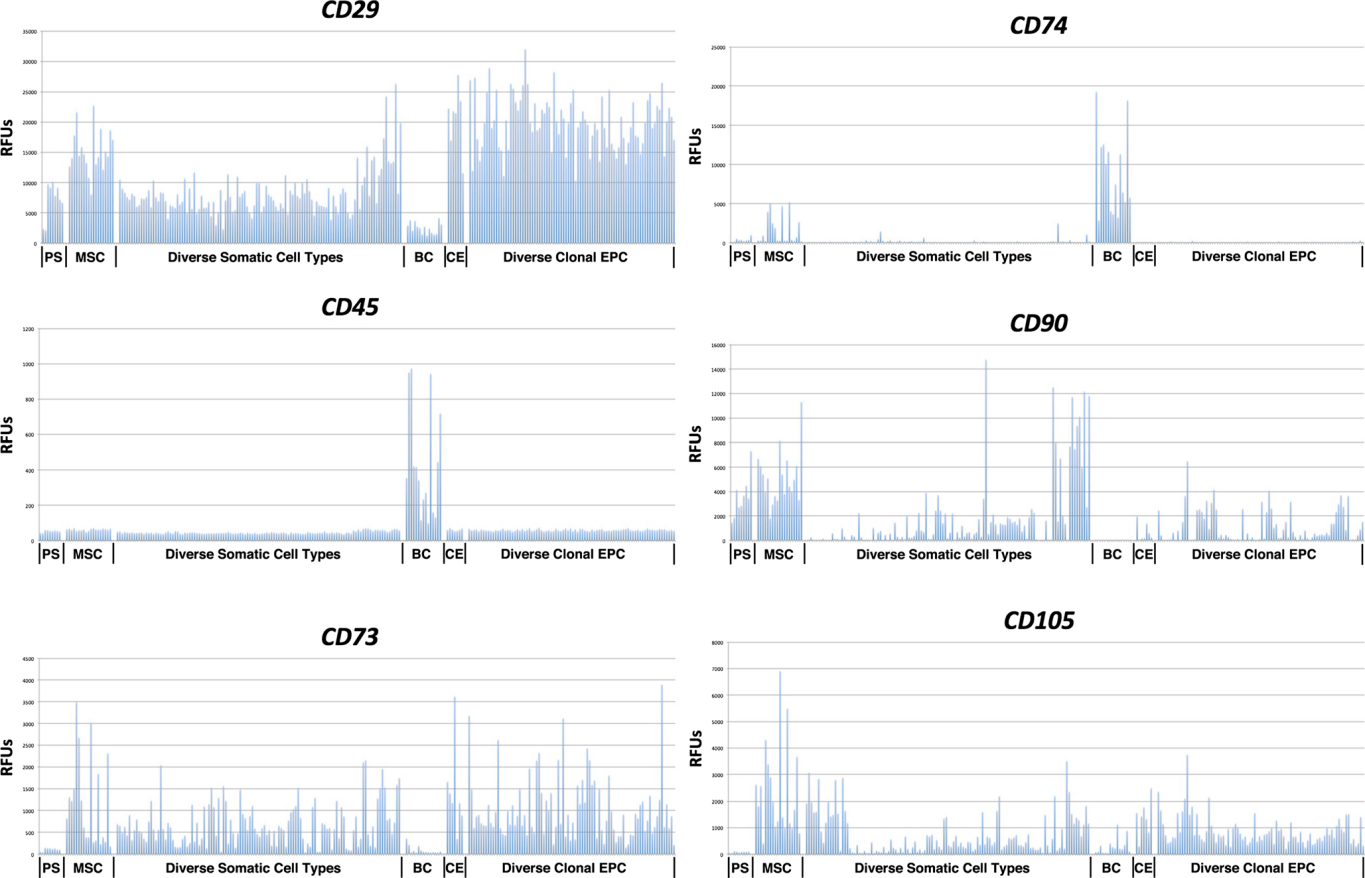
Expression levels of common MSC CD antigen markers in hPSC, adult stromal, blood, and embryonic progenitor cell types. The levels of expression (in relative fluorescence units (*RFUs*)) of the marker genes *CD29*, *CD45*, *CD73*, *CD74*, *CD90*, and *CD105* are shown. *PS* human pluripotent (embryonic) stem cells; *MSC* adult bone marrow-derived mesenchymal stem cells, *diverse somatic cell types* 111 fetal or adult-derived epithelial and mesenchymal cell types, *BC* 15 diverse blood cell types, *CE* seven hPSC-derived clonal embryonic progenitor cell lines previously shown to have osteochondral differentiation potential similar to adult MSCs, and 86 other diverse clonal embryonic progenitor (*EPC*) lines (used with permission from *Regen. Med.* (2013) **8**(2), 125–144) [21•]

It has been reported that the antigen CD74 is capable of distinguishing bone marrow MSCs from other types of mesenchymal cells [24]. As shown in Fig. 1, *CD74* is indeed expressed on an RNA level in definitive MSCs, but largely absent from the diverse hPSC-derived hEP cell lines, even those with a robust potential for chondrogenic differentiation. Given the lack of *CD74* expression in the majority of the clonal isolates, it appears likely that many published reports of MSCs derived from hPSCs should not be considered as meeting the most stringent criteria of true MSCs unless, at minimum, they were shown to express CD74. Many of these still-uncharacterized embryonic mesenchymal cell types may indeed have cellular fates and functionality that distinguishes them from MSCs. For example, we observed that clonal EP cell lines capable of osteochondral fates differentiate into cartilage lacking markers of hypertrophy such as *COL10A1* and *IHH* [25–27], the former being the most abundant transcript in adult bone marrow-derived MSCs and a marker of cells destined to promote vascular invasion rather than forming definitive chondrocytes such as those residing in the hyaline articular cartilage [28]. In this context, hPSC-derived EP lines (that do not differentiate to hypertrophic chondrocytes) may be better therapeutic candidates than MSCs (which are capable of differentiating to hypertrophic chondrocytes) for repairing damaged joints with cartilage having the needed mechanical properties for weight bearing joints. In addition, given the typically long initial telomere length of many hPS cells, the proliferative lifespan while retaining differentiation potential of the hEP cell lines can exceed that of adult-derived MSCs [25]. Therefore, we suggest that future studies of hPSC-derived mesenchymal cells should explore the unique and diverse properties of embryonic anlagen rather than assuming they are functionally identical to adult-derived MSCs. In addition, we suggest that, unless a precise association with specific anlagen such as forelimb bud mesenchyme or mandibular neural crest mesenchyme can be established, the hPSC-derived heterogeneous mesenchymal cultures be designated as “human embryonic fibroblasts,” instead of MSCs, in the same way that heterogeneous mouse embryo-derived mesenchyme is referred to as mouse embryonic fibroblasts (MEFs) and not MSCs.

## Conclusion

The complexity of the differentiated fate space of human pluripotent stem cells challenges current differentiation protocols. This is particularly the case when the intended use is for human clinical application. The demonstration that many heterogeneous cultures of mesenchymal cells from hPSC contains a diversity of up to 140-fold diversity out of 202 clonal lineages, combined with the recognition that many of these cell types have very different fates than bone marrow MSCs such as documented neural crest lineages capable of meningeal and choroid plexus differentiation, should cause concern when the intent is to generate the equivalent of adult-derived MSCs for human transplantation [5]. The hPSC-derived counterparts may, therefore, have very different fates, efficacy, and safety profiles.

It seems likely, therefore, that future trends in the field will need to include new and improved differentiation protocols that yield a higher resolution of the definition of the precise cell types being produced as well as where in the time course of embryological development the cells most closely correspond. Also critical, in our opinion, will be a global map of the transcriptome of human development with a cellular level of resolution. This cell ontology tree, similar to that being built at LifeMap Discovery [29] (*discovery.lifemapsc.com*), when properly correlated with the corresponding transcriptome of each cell type, may lead to a deeper understanding of the complex cascade of molecular development as well as a means of reproducibly manufacturing more precisely defined cellular therapeutics.

## Abbreviations

CFU-EP: Colony-forming unit-embryonic progenitor
CFU-F: Colony-forming unit-fibroblast
CNS: Central nervous system
FACS: Fluorescence activated cell sorting
GMP: Good manufacturing practices
hEP cells: Human embryonic progenitor cells
hES cells: Human embryonic stem cells
hPSCs: Human pluripotent stem cells
LMD: LifeMap Discovery
MSCs: Mesenchymal stem cells
NMF: Non-negative matrix factorization
RFUs: Relative fluorescence units
